# Children’s perception of food parenting practices: adaptation and validation of the comprehensive feeding practices questionnaire in Chilean adolescents

**DOI:** 10.3389/fpubh.2024.1343623

**Published:** 2024-03-12

**Authors:** Carola Del Valle, Horacio Miranda, Ligia Orellana, Klaus G. Grunet, Cristian Adasme-Berrios, Berta Schnettler

**Affiliations:** ^1^Doctorado en Ciencias Agroalimentarias y Medioambiente, Facultad de Ciencias Agropecuarias y Medioambiente, Universidad de La Frontera, Temuco, Chile; ^2^Facultad de Ciencias Agropecuarias y Medioambiente, Universidad de La Frontera, Temuco, Chile; ^3^Centro de Excelencia en Psicología Económica y del Consumo, Universidad de La Frontera, Temuco, Chile; ^4^Departamento de Psicología, Facultad de Educación, Ciencias Sociales y Humanidades, Universidad de La Frontera, Temuco, Chile; ^5^MAPP Centre, Aarhus University, Aarhus, Denmark; ^6^University of Vaasa, Vaasa, Finland; ^7^Departamento de Economía y Administración, Universidad Católica del Maule, Talca, Chile; ^8^Scientific and Technological Bioresource Nucleus (BIOREN-UFRO), Universidad de La Frontera, Temuco, Chile; ^9^Universidad Católica de Santiago de Guayaquil, Guayaquil, Ecuador

**Keywords:** CFPQ-Teen, parental feeding practices, adolescents, multigroup invariance, adolescent eating habits, psychometrics properties, validity, factor scores

## Abstract

**Introduction:**

Assessment of the Comprehensive Feeding Practices Questionnaire in adolescents (CFPQ-Teen) is still limited, with no evaluation of the measurement invariance. The participants comprised 473 Chilean adolescents of both sexes from dual-income nuclear families. The aims of this study were: (1) to adapt to Spanish and validate a model of five-factor version the CFPQ-Teen; (2) to examine the psychometric properties, (3) to evaluate the measurement invariance according to the adolescents’ gender; and (4) to compare the scores of each factor between female and male adolescents.

**Methods:**

The instrument was translated, back-translated, and adapted from the CFPQ-Teen, confirming the equivalence, conceptual, and face validity in a pilot sample of 40 adolescents. An exploratory factor analysis was performed on the five-factor model of the CFPQ-Teen: Monitoring, Adolescent Control, Restriction for weight control, Parental Modeling, and Environment. The Environment factor was eliminated as a result.

**Results:**

The confirmatory factor analysis presented good reliability, convergent, discriminant, and concurrent validity values. In addition, medium to high goodness-of-fit levels were obtained by eliminating an item from the Adolescent Control factor. These results confirm a final 20-item model representing four factors. The multigroup invariance analysis of the measurement model verified configural, metric, scalar, and partial strict invariance. No significant differences were found between females and males in the scores on the four factors.

**Discussion:**

These results enable comparisons by sex on the perceptions of Food Parenting Practices from the analyzed factors, primarily within the context of the Chilean sample.

## 1 Introduction

Obesity is a pandemic affecting all age groups (1) and is one of the most significant health issues today (2). Obesity has increased rapidly worldwide in recent decades, with a growing increase among adolescents (3). In Latin America, obesity rates have reached worrisome levels (4), with an increasing rate since 2016 estimated to reach 19.8% in 2030 (5). The prevalence of overweight and obesity is even more concerning in Chile (2), Ávila-Alpirez et al. (3) report that four out of every 10 adolescents between 12 and 19 years are overweight or obese.

Adolescence is characterized by complex transitions due to important physical but also psycho-social and dietary changes (6, 7). During adolescence, maladaptive eating behaviors (8) and disorganized eating habits can occur (9), which lead to a reduction in eating quality (6, 10, 11), and which are associated with weight gain (12–14) and obesity, which generally persists into adulthood (9, 15–18). Papamichael et al. (19) showed that adolescents adopt diets characterized by high levels of sugar, fats, processed meats, and salt, which have been linked to an increased risk of developing non-transmissible diseases sooner than usual (14, 17). This is a significant public health issue in all parts of the world (7, 20) given the associated negative consequences, which lead to a reduction in quality of life in the subsequent developmental stages (17, 21–24).

Negative eating behaviors during adolescence can be explained partly by the greater autonomy and independence acquired at this life stage (7, 23). However, although children acquire greater autonomy in adolescence (9), parents continue to be responsible for meal preparation and eating at home (7). Therefore, parents continue to play an important role in their children’s development of eating habits during this period (25, 26). This influence is supported by Family Systems Theory, which states that individuals involved in reciprocal relationships (such as parents and their children) can influence each other’s thoughts, emotions, behaviors (27), and eating habits [e.g., (28, 29)].

To instill healthy eating habits and avoid weight gain, parents can use different Food Parental Practices (FPP), i.e., specific feeding habits that parents use to influence what, when, and how much their children eat (30), both during and between family meals (31). Vaughn et al. (32) propose a classification of FPP into three categories: coercive control (e.g., restriction, pressure to eat, threats, and bribes), structure (e.g., modeling, monitoring, availability, and accessibility of foods, unstructured practices), and autonomy support (e.g., nutritional education, stimuli, reasoning, and negotiation). Studies on FPP have focused mainly on preschool children and early childhood (20, 33, 34), likely due to the evidence indicating that unhealthy eating habits in early childhood could lead to complex eating disorders in more advanced life stages (35) and that in 90% of cases, obesity in childhood remains in adolescence (36). However, this focus on early childhood has led to a paucity of studies on FPP in adolescence, particularly in Latin America (7, 37).

Nevertheless, the sparse evidence available had showed that FPP strongly influence eating preferences, food choice, and intake during adolescence (38, 39). In addition, recent studies have shown that different FPP applied to adolescents can have different impact, with modeling having a positive impact on healthy eating habits (23, 40), while other FPP such as restriction are associated with negative results, such as weight gain (30).

Several instruments are currently used to measure FPP in different age groups. One frequently used measure is the Comprehensive Feeding Practices Questionnaire (CFPQ), initially developed by Musher-Eizenman and Holub (41), which consists originally of 49 items representing 12 factors to measure child FPP. Later, Melbye et al. (42) adapted this instrument to be applied to parents of adolescents.

The psychometric properties of the CFPQ have been widely evaluated in different studies, which has led to proposals to reduce the number of factors (7, 42–48). For instance, studies conducted with the CFPQ (41), in Brazil in samples of parents of school-aged children (45, 48), and in Greece with a sample of parents with children between 2 and 12 years (46), reported a model of only six of the 12 original factors. The factors that have shown greater stability in previous studies with parents are Monitoring [e.g., (13, 30, 43)], Control [e.g., (43)], Restriction for weight control [e.g., (43, 49–51)], Modeling [e.g., (43, 52, 53)], and Environment [e.g., (13, 51)].

Piccoli et al. (7) then adapted this instrument to measure the perception that adolescents have of FPP exerted by their parents in a sample of Brazilian adolescents. These authors thus proposed a 10-factor model for the CFPQ-Teen, validating the CFPQ-Teen using factorial analysis. Nevertheless, there remain research gaps regarding the CFPQ that this study seeks to fulfill. Namely, this scale has not been adapted to Spanish, and its psychometric properties have not been assessed in other contexts. Therefore, the focus of this study was to adapt the CFPQ-Teen to Spanish and to evaluate the psychometric properties of a five-factor model of the CFPQ-Teen, considering those factors that have shown greater stability in previous studies with samples of parents.

Having a validated instrument that measures the perceptions of adolescents about the application of the FPP is relevant because of the discrepancies found in the reports of parents and adolescents. Adolescents provide a different account of what their parents do to influence their eating habits, compared to what the parent’s report, especially in the Latin American Spanish-speaking context, which presents significant increases in the rate of overweight and obesity in adolescents (3–5). Furthermore, adolescence represents the last life stage in which parents have the possibility of a greater influence on their children’s eating habits through FPP (54).

Another aspect little studied in Latin America is the differences in adolescents’ perceptions of FPP according to gender (55). Making comparisons between the perceptions of samples of female and male adolescents is relevant because the evidence indicates that females have healthier eating habits than males, which suggests that gender is an important factor in food choices (55). It has been shown that, in particular, male adolescents consume more meat and fewer vegetables, and female adolescents select healthier foods like fruits and vegetables (56).

According to Cortez et al. (57), the differences in eating habits according to gender in adolescence are associated with the ideal body stereotypes that society imposes. Adolescent females place excessive significance on being thin, viewing it as a sign of perfection and attractiveness and adopting a thin esthetic model that places enormous societal pressure on girls as they develop their body image and self-esteem. Conversely, males seek an ideal muscular mesomorph body (58). However, to the best of the authors’ knowledge, there are no studies available that have assessed measurement invariance of the CFPQ-Teen according to the gender of the adolescents, which enable a correct comparison of the perceptions that female and male adolescents have of the FPP their parents exercise to promote adequate nutrition and prevent weight gain. Therefore, it is necessary to verify the equivalence of the perceptions of female and male adolescents regarding the FPP exercised by their parents.

In summary, the novelty of this study lies in the adaptation of the CFPQ-Teen to Spanish, validate it, assess its psychometric properties, and estimate the measurement invariance according to the adolescents’ gender in a Latin American country. It should be highlighted that the CFPQ-Teen measure the adolescent’s perception of their parents’ FPP, which allows to broaden the vision of studies that only consider the perspective of parents. Having both the parents’ and adolescents’ perspectives would allow for the development of better intervention strategies to improve how parents exercise FPP with the goal of improving adolescents’ eating habits and health.

Based on this background, the aims of this study were: (1) to adapt and validate a five-factor model version of the CFPQ-Teen in Spanish; (2) to examine the psychometric properties, (3) to evaluate the measurement invariance according to the adolescents’ gender, and (4) to compare the scores of each factor between female and male adolescents.

## 2 Methods

### 2.1 Sampling and procedure

Non-probability sampling was used to recruit a sample of adolescents between 12 and 16 years of age. To obtain a suitable representation of different socioeconomic levels (high, middle, and low), participants were selected through proportional quota sampling and recruited through contact with the authorities at seven schools located in Temuco, Chile. The questionnaire was administered to 243 female and 230 male adolescents between July and December 2019. The total sample comprised 473 adolescents, which according to the criteria of Comrey and Lee (59), is a very good sample size. Because the sampling design was non-probabilistic, the sample size verification did not include sampling error, that was also confirmed through the estimation of the minimum sample size for models of structural equations models based on the RMSEA adjustment index according to Kim (60), MacCallum et al. (61), and Steiger et al. (62).

Prior to data collection, the parents of the adolescents were asked to sign a consent form to authorize the participation of the female or male adolescent. The adolescents were also asked to sign a consent form. The forms ensured their voluntary participation and protected the confidentiality and anonymity of the data collected. Then, the surveys were applied by trained interviewers (students in the final years of the Psychology program) to each adolescent personally, recording their answers on the online survey platform QuestionPro (QuestionPro Inc.), using tablets to reduce errors in the transcription of the responses. This study is part of a cross-sectional and longitudinal study that examines the interdependence between the job, family, and food domains in Chilean families. The adolescents’ family received a bank transfer of 15 USD as a thank you for their participation. The Universidad de La Frontera Ethics Committee approved the research protocol (Protocol Number 007/2019).

### 2.2 Adaptation of the instrument

The adapted instrument corresponded to the Comprehensive Feeding Practices Questionnaire for use on adolescents CFPQ-Teen validated by Piccoli et al. (7), which comprises 43 items representing 10 factors. The content adaptation of the model with five factors from the CFPQ-Teen included a group of experts who performed translation/back-translation process of the items between English and Spanish. In these items some substitutions were made of the word “caregiver” or “S/he” for “your parents” or “my parents,” and the replacement of some foods for similar products available in the Chilean market (Supplementary material). The adapted items were pilot tested with 40 adolescents comprising a representative sample in terms of the sex and age of the target population. The result was a translated and adapted version of the questionnaire, as well as obtaining the conceptual equivalence (63) and face validity (64) of the factors Monitoring, Adolescent Control, Restriction for weight control, Parental Modeling, and Environment on the CFPQ-Teen, and understanding and clarity were verified. The face validity obtained in the initial stage of the instrument’s adaptation is a preliminary measurement of validity because it is subjectively evaluated without statistical tests (64). This process entails assessing the ease of item interpretation (65), but it does not constitute a quantitative validity *per se*. Quantitative validity is assessed in subsequent stages when the psychometric properties of the instrument are evaluated (66).

### 2.3 Instruments

The applied instrument corresponded to the CFPQ-Teen (7). The selection of the number of factors to be used in this study is based on the reduction and validation of the CFPQ instrument carried out in Chile by Del Valle et al. (43). The adolescents responded the five-factor model the CFPQ-Teen: Monitoring, which assesses the adolescents’ perception of the frequency with which parents monitor the consumption of unhealthy foods; Adolescent Control, which assesses the frequency at which parents are more permissive regarding food behavior and habits in adolescents; Restriction for weight control, assesses the adolescents’ perception of parental control over food intake to decrease or maintain their weight; Parental Modeling, assesses how adolescents perceive their parents as a model or reference for them regarding healthy food habits; and Environment, measures the availability of healthy foods at home. Obtained the following Cronbach’s alphas: Monitoring = 0.85, Adolescent Control = 0.67, Restriction for weight control = 0.83, Parental Modeling = 0.82, and Environment = 0.64.

The Adolescent Control and Monitoring items were answered on a five-point Likert-type scale, from 1: “never” to 5: “always.” The remaining factors, Restriction for weight control, Parental Modeling, and Environment, were answered on a five-point Likert-type scale, from 1: “totally disagree” to 5 “totally agree.”

To evaluate the concurrent external validity, the Alternative Healthy Eating Index (AHEI) was used as the manifest dependent variable, which is an adaptation of the US-HEI (67) developed by Norte and Ortiz (68) to measure dietary quality in Spanish-speaking populations. Participants were asked to indicate the consumption frequency of nine food groups: (1) Cereals and derivatives; (2) Vegetables; (3) Fruit; (4) Milk and milk products; (5) Meats; (6) Legumes; (7) Sausages and processed meats; (8) Sweets, and (9) Sugary beverages. Data on the consumption frequency of each food group became a score from 0 to 10 according to the degree of fulfillment of dietary recommendations (68). A dietary variety score was calculated for each respondent, considering the fulfillment of each of the daily and weekly recommendations. The AHEI score was calculated by adding the score obtained in each variable. The scores from the different food groups and components total 100 points. Scores over 80 are indicative of “a healthy” diet; scores between 51 and 80 are a diet that “needs changes”; scores below 50 are “unhealthy” diets (67). In addition, although the AHEI may be a useful tool to measure diet quality, this measure does not include all possible food groups and the quantity of food consumed is not assessed. This version has been used recently in previous studies in Chile to assess the dietary quality in adolescents [e.g., (28, 29)].

The questionnaire includes questions about the sociodemographic classification of the participants and their families (Table 1).

**Table 1:** Sample characteristics: Mean, (*SD*), percentage (%), *p* value.

Variable analyzed (*n* = 473)	Value	*p* value
Age [Mean *(SD)*]		
Mothers	39.1 (7.2)	
Fathers	42.0 (8.9)	0.0001^1^
Socioeconomic status (%)		
High	22.2	
Middle	61.5	
Low	16.3	0.0001^2^
Employed (%)		
Mothers	72.7	
Fathers	74.8	0.461^3^
Full time workers (%)		
Mothers	59.2	
Fathers	72.3	0.0001^3^
Income [Mean *(SD)*]		
Mothers	508,821 (458,151)	
Fathers	739,594 (775,829)	0.0001^1^
Adolescent’s gender (%)		
Female	48.6	
Male	51.4	0.232^3^
Number of children [Mean *(SD)*]	2.2 (0.8)	
Adolescent’s age [Mean *(SD)*]	12.5 (1.7)	

^1^Significance of the Student *t*-test for independent samples, ^2^Significance of the Chi-Square goodness-of-fit-test, and ^3^Proportions comparison test.

### 2.4 Data analysis

A cross-sectional, non-experimental design was used in this study. The Statistics Package for Social Sciences (IBM SPSS) v. 23 was used for the descriptive analysis, skewness and kurtosis indices, and comparison of the scores on the factors between males and females. In addition, the SPSS macro for the Solomon method was used (69) to divide the sample into estimation and validation samples. Each sample contained approximately 50% of the participants (70) to be able to use an estimation sample (*n* = 236) in the exploratory factor analysis (EFA) and a validation sample (*n* = 237) in the confirmatory factor analysis (CFA).

The Mplus software v. 8.4 was used for the EFA (71), CFA (72), and the multigroup invariance analysis of the measurement model (73) between the female and male samples.

To estimate the loadings in the EFA, the weighted least squares means and variance adjusted method (WLSMV) was used (74) in the five factors from the CFPQ-Teen. The polychoric correlation matrix was used (75) due to the ordinal response scale of the items (76, 77). The homogeneity of the items and the number of factors to be retained were verified by Horn’s parallel analysis (78). Sample adequacy was evaluated using the Kaiser-Meyer-Olkin index >0.7, a value of the correlation matrix determinant >0.0, and a significant Bartlett’s test of sphericity *p* < 0.001, which determined the relevance of applying the EFA to the empirical correlation matrix of the items.

The WLSMV method was also used for the CFA estimations (74). Once the measurement model had been confirmed with the CFA, the goodness-of-fit was estimated, as well as the following psychometric indices: construct validity through convergent validity, discriminant validity, external validity through concurrent validity, and reliability. The convergent validity was determined by the average variance extracted (AVE) of each factor of the CFPQ-Teen, which must present a value >0.50 (79). In addition, according to (80), it is expected that the items are statistically significant *p* ≤ 0.05 and that, ideally, they present standardized factor loadings >0.50. The discriminant validity was verified with Fornell and Larcker’s method (81, 82) in which the squared correlations must be smaller than the AVE of the associated factors. Finally, concurrent external validity was verified by the statistical significance (*p* ≤ 0.05) of the regression coefficients or model trajectories (PATH) of the CFPQ-Teen to the AHEI (68) manifest variable.

The reliability was estimated using McDonald’s omega coefficient (83). Next, the measurement invariance analysis of the resulting model of the CFA was performed. The sequential procedure of the invariance measurement model consists of the evaluation of the configural (0), metric (1), scalar (2), and strict (3) models.

To evaluate the fulfillment of the configural (0) model, the overall fit index of the root mean square error of approximation (RMSEA) (82), the comparative fit index (CFI), and the Tucker-Lewis index (TLI) (84) were used. It is considered a good fit when RMSEA ≤0.06, CFI and TLI ≥ 0.95 (84) and an acceptable fit if RMSEA ≤0.08, CFI and TLI ≥ 0.90 (85). In addition, the Chi-Square difference test “DIFF TEST” was used for the statistical significance test between the sequential invariance models (86) for nested models. Finally, partial invariance was calculated when the fulfillment of invariance was not verified in some of the stages.

The fulfillment of the multigroup invariance of the measurement model made it possible to compare the scores from the factors on the CFPQ-Teen between females and males. Given that the items correspond to an ordinal scale, the non-normal distribution of their total was corroborated using the Kolmogorov–Smirnov test with the Lilliefors correction (87). These comparisons were made using the Mann–Whitney U non-parametric test (88).

## 3 Results

### 3.1 Exploratory factor analysis

Horn’s parallel analysis determined a dimensionality of four retained factors for the 21 items of the CFPQ-Teen five-factor model. The Kaiser-Meyer-Olkin (KMO) measure of sample adequacy was 0.081, determinant >0.0, and the Bartlett’s test of sphericity was significant (*p* < 0.001).

The factor Environment had two negative cross-loadings with the factor Adolescent Control. In addition, two of the items in Environment showed greater loadings with the factor Parental Modeling: item 21, “During meal times at home, there are several healthy food items available for me to eat,” and item 14, “Most of the food at home is healthy.” Thus, the factor Environment was comprised only of two inverse items: “At home, there is a lot of snacks (french fries, potato sticks, tortilla chips, and salty popcorn)” and “At home, there is a lot of sweets (ice cream, desserts, candies, and cake),” describing the presence of unhealthy food in the home (Table 2). Therefore, the factor Environment was eliminated in this study due to its lack of homogeneity, resulting in a measurement model with four factors: Monitoring, Adolescent Control, Restriction for weight control, and Parental Modeling.

**Table 2:** Exploratory factor analysis of five-factor CFPQ-Teen model.

Items	Monitoring	Control	Restriction	Modeling	Environment
Monitoring 2	**0.959**	−0.050	−0.032	−0.037	−0.001
Monitoring 1	**0.955**	−0.079	−0.039	0.049	−0.139
Monitoring 3	**0.796**	0.078	0.173	−0.002	0.105
Monitoring 4	**0.702**	0.020	0.212	0.026	0.127
Control 6	0.087	**0.647**	−0.011	0.105	−0.142
Control 10	0.039	**0.644**	0.140	−0.010	−0.017
Control 5	−0.074	**0.565**	−0.220	0.025	**−0.322**
Control 1	−0.090	**0.514**	−0.191	−0.025	**−0.355**
Control12	0.044	**0.485**	0.027	−0.149	0.075
Restriction 24	−0.030	0.019	**0.854**	−0.078	−0.012
Restriction 29	−0.035	−0.013	**0.844**	−0.008	−0.047
Restriction 26	−0.045	−0.071	**0.833**	0.095	−0.144
Restriction 31	0.024	0.033	**0.798**	−0.061	0.211
Restriction 30	0.004	0.006	**0.797**	0.046	0.201
Restriction 39	0.078	−0.113	**0.643**	0.003	−0.034
Restriction 36	0.063	0.061	**0.615**	0.033	0.035
Restriction 18	0.183	−0.250	**0.391**	0.133	−0.053
Modeling 42	−0.031	−0.001	0.039	**0.858**	0.058
Modeling 41	0.031	0.103	0.108	**0.779**	0.026
Modeling 38	−0.006	0.010	0.034	**0.774**	0.045
Modeling 40	0.026	0.027	0.031	**0.653**	0.166
Environment 16	0.027	−0.017	−0.121	0.024	**0.782**
Environment 32	−0.180	−0.057	−0.043	0.098	**0.739**
Environment 14	−0.001	−0.258	−0.099	**0.630**	−0.022
Environment 21	0.062	−0.141	−0.071	**0.597**	−0.112

Original CFPQ-Teen: Monitoring, Control = Adolescent Control; Restriction = Restriction for weight control; Modeling = Parental Modeling and Environment. The wording of each item is found in the Supplementary material. Bold values indicate higher factor loadings for each factor.

### 3.2 Confirmatory factor analysis

In the CFA, item 12 of the factor Adolescent Control was eliminated: “Your parents allow you to leave the table when you are satisfied, even if others have not finished eating?” because it presented a factor loading <0.4. The measurement model had an acceptable overall fit (RMSEA = 0.080) and good incremental fit indexes (CFI = 0.965, TLI = 0.959) (Table 3). The reduced measurement model with four factors from the CFPQ-Teen lived up to the requirements for convergent, discriminant, and concurrent external validity. The convergent validity of each factor was confirmed by the AVE ≥ 0.50 (89); Monitoring = 0.80, Adolescent Control = 0.50, Restriction for weight control = 0.61, and Parental Modeling = 0.65. Discriminant validity according to Fornell and Larcker’ method was established because the squared correlations were smaller than the AVE of each factor: Monitoring and Adolescent Control = 0.15, Monitoring and Restriction for weight control = 0.20, Monitoring and Parental Modeling = 0.14, Adolescent Control and Restriction for weight control = 0.11, Adolescent Control and Parental Modeling = 0.08, Restriction for weight control, and Parental Modeling = 0.13.

**Table 3:** Confirmatory factor analysis of four-factor CFPQ-Teen model.

Items	Monitoring	Control	Restriction	Modeling
Monitoring 2	0.956			
Monitoring 1	0.906			
Monitoring 3	0.854			
Monitoring 4	0.830			
Control 11		0.797		
Control 5		0.779		
Control 6		0.612		
Control 10		0.516		
Restriction 26			0.882	
Restriction 30			0.877	
Restriction 31			0.860	
Restriction 29			0.832	
Restriction 24			0.824	
Restriction 36			0.793	
Restriction 18			0.703	
Restriction 39			0.691	
Modeling 41				0.909
Modeling 42				0.847
Modeling 40				0.742
Modeling 38				0.737

Original CFPQ-Teen: Monitoring, Control = Adolescent Control, Restriction = Restriction for weight control, and Modeling = Parental Modeling.

The wording of each item is found in the Supplementary material.

Concurrent external validity of the reduced CFPQ-Teen model with regard to the AHEI was confirmed by the PATH, which was statistically significant (*p* < 0.05) with the following coefficients: Monitoring = 0.100, Adolescent Control = −0.253, Restriction for weight control = −0.140, and Parental Modeling = 0.236. It is noteworthy that Adolescent Control and Restriction for weight control had an inverse relation with the AHEI (Table 4).

**Table 4:** Convergent, discriminant, and concurrent validity of four-factor CFPQ-Teen model.

Factor	Monitoring	Control	Restriction	Modeling	Min λ	Max λ	PATH	*p*(PATH)
Monitoring	0.80	0.066	0.180	0.087	0.830	0.956	0.100	0.039
Control	−0.257	0.50	0.057	0.043	0.516	0.797	−0.253	0.001
Restriction	0.424	−0.239	0.61	0.152	0.691	0.882	−0.140	0.011
Modeling	0.295	−0.208	0.391	0.65	0.737	0.909	0.236	0.001

Diagonal Correlation Matrix = AVE (>0.50).

Upper diagonal = Squared root of correlation coefficient (lower than AVE of factors).

Lower diagonal = Polychoric Correlation Coefficient (correlation coefficient for ordinal variables).

Min λ; Max λ = Minimum and maximum loading (0.3 < λ < 1.0).

PATH = Regression coefficient.

*p*(PATH) = *p* value of the PATH.

The reliability was middle to high as the McDonald’s omega coefficient for each factor of the reduced model were: Monitoring = 0.91, Adolescent Control = 0.69, Restriction for weight control = 0.90, and Parental Modeling = 0.83.

### 3.3 Multigroup invariance analysis of the measurement model

The fit of the four-factor measurement model was: χ^2^(df) = 369.911 (164), *p* ≤ 0.001, RMSEA = 0.074; CFI = 0.966; TLI = 0.960 in the female sample, and χ^2^(df) = 460.616 (164), *p* ≤ 0.001, RMSEA = 0.086; CFI = 0.964; TLI = 0.958 in the male sample. All the skewness and kurtosis indexes showed non-normal skewed distributions (Table 5).

**Table 5:** CFA, standardized loadings, and descriptive characteristics of female and male samples.

	Females adolescents^a^ (*n* = 230)					Male adolescents^b^ (*n* = 243)					
Factors and items	Loadings	M	SD	Skewness	Kurtosis	Items	Loadings	M	SD	Skewness	Kurtosis
Monitoring											
Monitoring 2	0.930	3.40	1.31	−0.43	−0.90	2	0.953	3.50	1.32	−0.55	−0.78
Monitoring 1	0.900	3.45	1.28	−0.53	−0.75	1	0.937	3.57	1.29	−0.56	−0.81
Monitoring 3	0.854	3.41	1.24	−0.45	−0.70	3	0.884	3.42	1.28	−0.36	−0.96
Monitoring 4	0.837	3.45	1.35	−0.42	−1.03	4	0.858	3.54	1.33	−0.50	−0.92
Adolescent control											
Control 5	0.904	1.90	1.12	1.19	0.70	11	0.860	1.90	1.13	0.12	0.70
Control 11	0.793	2.84	1.11	0.14	−0.46	5	0.741	2.78	1.11	0.19	−0.52
Control 6	0.431	2.63	1.22	0.26	−0.81	6	0.541	2.53	1.26	0.46	−0.71
Control 10	0.299	2.54	1.07	0.18	−0.05	10	0.468	2.37	1.54	0.53	−0.08
Restriction for weight control											
Restriction 30	0.879	3.33	1.47	−0.41	−1.22	26	0.871	3.27	1.37	−0.39	−1.05
Restriction 29	0.874	2.70	1.55	0.22	−1.48	31	0.870	3.24	1.54	−0.32	−1.38
Restriction 24	0.834	3.15	1.27	−0.29	−0.86	30	0.849	2.87	1.45	−0.02	−1.39
Restriction 26	0.823	3.18	1.52	−0.31	−1.35	24	0.779	2.29	1.36	−0.60	−0.98
Restriction 31	0.809	3.13	1.45	−0.25	−1.30	29	0.746	3.41	1.24	−0.39	−0.73
Restriction 36	0.705	3.11	1.50	−0.23	−1.41	36	0.734	3.28	1.42	−0.41	−1.13
Restriction 39	0.697	2.59	1.36	0.32	−1.07	18	0.685	3.42	1.43	−0.57	−0.99
Restriction 18	0.642	2.07	1.31	0.95	−0.32	39	0.675	2.82	1.40	0.05	−1.25
Parental modeling											
Modeling 41	0.881	3.86	1.21	−0.13	0.27	41	0.878	4.07	0.98	−0.90	0.27
Modeling 42	0.824	4.00	1.03	−0.63	−0.33	42	0.875	3.82	1.23	−0.82	−0.33
Modeling 40	0.743	3.53	1.17	−0.59	0.26	38	0.843	3.81	1.13	−0.92	0.26
Modeling 38	0.662	3.74	1.07	0.03	−0.13	40	0.736	3.91	1.09	−0.76	−0.13

M, Mean; SD, Standard deviation. The wording of each item is found in the Supplementary material.

Model 0, or the configural model, showed an acceptable fit (RMSEA = 0.080; CFI = 0.965; TLI = 0.959), confirming the configural invariance of the instrument. The DIFF TEST between model 0 configural model and model 1 metric [χ^2^(15) = 17.328, *p* = 0.365] confirmed the metric invariance. The DIFF TEST between Model 1 metric and Model 2 scalar [χ^2^(56) = 69.468, *p* = 0.107] confirmed the scalar invariance.

The DIFF TEST for strict invariance showed a significant difference [χ^2^(20) = 48.899, *p* = 0.0003]. As a result, partial strict invariance 1 released the residual of item 38 from the factor Modeling, “My parents eat healthy food to give me an example of healthy eating habits” and the partial strict 2 released the residual of item 29 from the factor Restriction for weight control, “If I eat more than normally at one meal, my parents reduce the quantity of food at the next meal” to achieve the equivalence of goodness-of-fit between the correlation matrix predicted by the measurement model and the empirical correlation matrix of the observed data. Thus, it was possible to fulfill the partial 2 invariance with a DIFF TEST [χ^2^(18) = 24.926, *p* = 0.127] with good incremental fit indices (CFI = 0.968; TLI = 0.971; RMSEA = 0.068) (Table 6).

**Table 6:** Multigroup measurement model invariance analysis with ordinal categorical indicators variables.

Model estimator (WLSMV)	χ^2^	df	D_DIFF TEST	D_df	p_DIFF TEST	RMSEA	CFI	TLI
Model 0: Configural invariance (all loadings and thresholds and residuals are freely estimated)	828.000	212	─	─	─	0.080	0.965	0.959
Model 1: Metric invariance (loadings are fixed across groups, thresholds and residuals are freely estimated)	836.608	196	17.328	15	0.365	0.078	0.966	0.922
Model 2: Scalar invariance (loadings and thresholds are fixed across groups and residuals are freely estimated)	881.261	140	69.468	56	0.107	0.071	0.966	0.968
Model 3: Strict invariance (loadings, thresholds, and residuals are fixed across groups)	903.012	120	48.899	20	0.0003	0.070	0.966	0.969
Model 3P1: Partial strict invariance 1 (loadings and thresholds are fixed across groups, with residual variance of item modeling 38 being freely estimated)	821.921	121	37.269	19	0.007	0.069	0.967	0.970
Model 3P2: Partial strict invariance 2 (loadings and thresholds are fixed across groups, with residual variance of item modeling 38 and item restriction for weight control 29 being freely estimated)	880.663	122	24.926	18	0.127	0.068	0.968	0.971

χ2, Chi-Square; df, Degrees of freedom; D DIFF-TEST, Delta of DIFF-TEST between goodness-of-fit test a model and previous model; D df, Delta of DIFF-TEST degrees of freedom; p DIFF-TEST, Statistical significance of Delta of DIFF-TEST; RMSEA, Root mean square error of approximation; CFI, Comparative fit index; and TLI, Tucker-Lewis.

Additional indicators for changes in RMSEA, CFI, and TLI showed that RMSEA started with an acceptable value of 0.08 within the range of 0.06–0.08 and decreased to 0.068 at the final stage of strict invariance. The CFI and TLI started with good levels of 0.965 and 0.959 and increased to 0.969 and 0.971, respectively, at the strict invariance stage. All indicators from loadings equivalence changed by 0.001 units between each subsequent stage of invariance.

Finally, the comparison of the scores of the four factors of the reduced CFPQ-Teen did not present any statistically significant differences (*p* > 0.05) between female and male adolescents (Table 7).

**Table 7:** Comparison of factors scores of four-factor CFPQ-Teen model between the female and male samples.

	Female				Male				
Factor	Mean	Median	LCI.95	UCI.95	Mean	Median	LCI.95	UCI.95	*p* (U-MW)
Monitoring	13.7	15	14	15	14.0	15	14	16	0.38
Control	9.9	10	9	10	9.6	9	8.8	10	0.20
Restriction	23.3	24	23	25	24.6	26	24	27	0.07
Modeling	15.1	15	15	16	15.6	16	16	17	0.08

*p* (U-MW), Significance of Mann–Whitney non-parametric test; LCI, Lower confidence interval; and UCI, Upper confidence interval.

## 4 Discussion and conclusion

Previous studies have focused mainly on measuring FPP in parents of preschoolers (20). In addition, there is a small number of studies that measure FPP in adolescents, particularly in Latin America (28, 29). One of the most frequently used instruments to measure FPP is the CFPQ (41), which is answered by parents. The need to validate this instrument cross-culturally led to Ángel et al. (90) adapting the CFPQ to Spanish in a sample of Mexican mothers. Meanwhile, Del Valle et al. (43) adapted and validated a Spanish five-factor model version of the CFPQ (42) to be answered by parents of adolescents in Chile, in addition to considering measurement invariance according to the parent’s gender. However, given the differences between what the parents and children state regarding FPP that the parent offer (91), it is necessary to consider the adolescents’ responded perceptions of the FPP that their parents exercise in the home. In response, Piccoli et al. (7) adapted the CFPQ to Portuguese and measured the perception of FPP in adolescents (CFPQ-Teen) with a sample of adolescents from 12 to 18 years of age in Brazil. These authors validated a model comprised of 43 items representing 10 factors.

In light of the considerable increase in the prevalence of overweight and obesity in Spanish-speaking Latin American adolescents (5) and the need to conduct research with a gender perspective (55), the present study focused on adapting and validating the version of CFPQ-Teen (7) in a Spanish-speaking Latin American country considering possible differences between female and male adolescents. Thus, to the best of the authors’ knowledge, this is the first study to integrate the adaptation of the CFPQ-Teen to Spanish, validate it, assess its psychometric properties, estimate the measurement invariance according to the adolescents’ gender, and compare the scores of the factors, in a reduced four-factor model selected based on the factor stability reported in previous studies (43–47).

The psychometric analysis of the four factors model of the CFPQ-Teen using the EFA led to the elimination of the factor Environment, because two of its items had high loadings with the factor Parental Modeling, such that only two inverse items were left. In other studies, this factor has also been shown to have problems with internal consistency and homogeneity (7, 42–45, 47, 48). Eliminating this factor is in accordance with the criterion of Fabrigar et al. (92), which recommends keeping a minimum of three to four items per factor evaluated and the general EFA criteria recommended by Streiner (93). As a result of the EFA, a reduced measurement model was reported with four factors: Monitoring with four items, Adolescent Control with five items, Restriction for weight control with eight items, and Parental Modeling with four items.

In the CFA, item 12 of the factor Adolescent Control, which had a loading <0.40: “Do your parents allow you to leave the table when you are satisfied even if others have not finished eating?,” was removed from the instrument. The removal of this item is in line with recent studies (42, 43, 46), because this item primarily reflects the rupture of social norms related to meals rather than the control of the child about what and when to eat (42).

An adapted and validated Spanish 20-item version was finally obtained, with four factors on the CFPQ-Teen to be answered by adolescents: Monitoring with four items, Adolescent Control with four items, Restriction for weight control with eight items, and Parental Modeling with four items. The evaluation of the psychometric properties of the resulting measurement model from the CFA showed acceptable to good goodness-of-fit levels and good convergent, discriminant, external concurrent validity and reliability, demonstrating that it is a valid instrument to assess adolescents’ perception of the factors studied here. Concerning external concurrent validity, the positive association between dietary quality and monitoring and modeling confirms previous studies, which indicate that monitoring and modeling promote healthier eating habits in adolescents (28, 29). Moreover, the negative relation between restriction and dietary quality confirms previous studies associating this FPP with negative results in adolescents’ eating habits (30). The negative association between the factor control and dietary quality confirms that parents controlling less what adolescents eat in response to their search for autonomy in food choices (23) leads to adolescents having reduced dietary quality (13). Therefore, it may be concluded that the adapted and validated Spanish version of the reduced CFPQ-Teen constitutes a valid and reliable instrument to evaluate the four factors studied in Chilean adolescents. Obtaining a reduced CFPQ-Teen in this study contributes to a better understanding and interpretation of FPP in adolescents.

Furthermore, the model showed measurement equivalence of the initial factor structure up to the levels of communality and uniqueness of the items. The results obtained in the measurement invariance analysis verified the fulfillment of the configural, metric, scalar, and partial strict invariance. This means that the CFPQ-Teen measures the perceptions of female and male adolescents of the FPP included in this investigation equally well.

The fulfillment of configural invariance showed that the number of factors and location of the items in the measurement model is equivalent in the female and male adolescent samples. The fulfillment of metric invariance showed that the instrument is equivalent in measuring the perception of the meaning of the factors in the female and male samples as evidenced by identical factor loadings. The fulfillment of scalar invariance verified that the instrument is equivalent in the levels of the five-point response possibilities of the Likert-type scale. When evaluating strict invariance, which measures the proximity between the values predicted by the measurement model and the data collected in the sample, two residuals had to be released to obtain its confirmation. First, the residual of item 38 from the Parental Modeling, “My parents eat healthy food to give me an example of healthy eating habits,” was released because it presented a different goodness-of-fit between the predictions and the empirical values of the correlation matrices. Eliminating the equivalence restriction between the two samples in item 38 confirmed partial strict invariance 1. Next, it was necessary to release the estimation of a second residual in item 29 of the factor Restriction for weight control, “If I eat more than normally at one meal, my parents reduce the quantity of food at the next meal,” because it presented a different goodness-of-fit between the residuals of the empirical correlation matrices and was reproduced by the measurement model between the two samples. The elimination of the equivalence restriction in item 29 confirmed partial strict invariance 2. These results can be associated with male adolescents presenting a greater frequency of high scores on the Likert-type response scale in both items, indicating that unlike females, males perceive that parents show them a greater consumption of healthy food and unlike females, males perceive receiving less food when their parents consider they have eaten more than normal at a previous meal. These results may be associated with parents employing more restrictive FPP with their children (94), which vary according to the perception of risk against diseases such as obesity (31).

Among the limitations of this study, it must be considered that the adolescents who participated belong to families in which both parents work, and they might have less time to worry about their adolescent children’s eating. A cross-sectional design and a medium-sized non-probability sample were used (*n* = 473). Furthermore, our sample is not representative of the Chilean population of adolescents because the sample only include adolescents between the ages of 12 and 16. In addition, this study was developed in a single Spanish-speaking Latin American country. Although the AHEI may be a useful tool to measure diet quality, this measure does not include all possible food groups and the quantity of food consumed is not assessed.

Future research should take into account probability samples of the population, cross-cultural studies, and longitudinal designs. In this regard, it would be feasible to gauge how FPP has changed over time, and it might be possible to achieve external validity by comparing this measure to a gold standard or external predictive validation using data observed in longitudinal studies. Future studies should also consider performing a profile transition analysis to examine latent heterogeneity over time. Future studies may also evaluate the measurement invariance according to another relevant characteristics of the adolescents, such as different levels of education or household income.

This is the first study to adapt a final model of the four-factor version of the CFPQ-Teen into Spanish, verifying the psychometric properties of reliability, validity. Moreover, to our knowledge, this is the only study to date that has assessed the measurement equivalency of the perception of male and female adolescents through multigroup invariance analysis of a measurement model. This analysis made it possible to measure and compare the scores of the factors on the CFPQ-Teen between female and male adolescent samples in a Spanish-speaking context. This study provides a unique, valid and reliable questionnaire to measure and assess the understanding of how female and male adolescents perceive and internalize four FPP exercised by their parents in the family context, making it possible to establish other associations with characteristics of the parents and the adolescents, such as age and socioeconomic level, among other sociodemographic variables.

Although adolescents show differences in eating habits, theoretically our results suggest that male and female adolescents have similar perceptions of their parents’ control, monitoring, modeling, and restriction for weight control. The findings from this study also have practical implications. The results allow to identify the FPP that require greater emphasis on the part of parents, as well as those FPP in which a higher level of mastery is present, from the perspective of their adolescent children. This research contributes to the construction of scenarios based on FPP factors for their application in research on eating behavior and its implications in the orientation and production of the food industry, as well as in health and educational organizations and institutions through different intervention programs applied to adolescents and their parents. This is relevant to promote an improvement in the eating habits of families with adolescent children.

## Data availability statement

The raw data supporting the conclusions of this article will be made available by the authors, without undue reservation.

## Ethics statement

The studies involving humans were approved by the Universidad de La Frontera Ethics Committee (Protocol Number 007/2019). The studies were conducted in accordance with the local legislation and institutional requirements. Written informed consent to participate in this study was obtained from the participants’ legal guardian/next of kin. The adolescents also provided their own written informed consent.

## Author contributions

CV: Data curation, Formal analysis, Methodology, Software, Writing – original draft, Writing – review & editing. HM: Data curation, Formal analysis, Methodology, Software, Writing – review & editing. LO: Investigation, Writing – review & editing. KG: Supervision, Writing – review & editing. CA-B: Supervision, Writing – review & editing. BS: Conceptualization, Funding acquisition, Investigation, Project administration, Resources, Supervision, Writing – review & editing.
