# Supplementary material for: Children’s perception of food parenting practices: adaptation and validation of the comprehensive feeding practices questionnaire in Chilean adolescents
Source: Front Public Health. 2024 Mar 12;12:1343623. doi: 10.3389/fpubh.2024.1343623 (PMC10972623; doi:10.3389/fpubh.2024.1343623)
Supplement: Supplementary file 1 [file Table_1.DOCX]

Supplementary Material

# Supplementary Table 1

**Adaptation of CFPQ-Teen (Piccoli et al., 2017) from English to Spanish.**

| **Factors** | **Items** |
| --- | --- |
| **Monitoring** | How often does this caregiver keep track of the quantity os sweets (or ice cream, cakes, pies, chocolates, candies) that you eat? (1) |
|  | ¿Con que frecuencia tus padres revisan la cantidad de dulces (o helados, pasteles, chocolates, caramelos, pies, queques) que comes? |
|  | How often does this caregiver Keep track of the quantity of industrialized snacks (potato chips, munchies, cheese pastries, etc.) that you eat? (2) |
|  | ¿Con qué frecuencia tus padres revisan la cantidad de snacks industrializados (papas fritas, ramitas, doritos) que comes? |
|  | How often does this caregiver keep track of the quantity of fatty foods (Hamburgers, snacks, mayonnaise, etc.) that you eat? (3) |
|  | ¿Con qué frecuencia tus padres revisan la cantidad de comida alta en grasa (hamburguesas, snacks, mayonesa) que comes? |
|  | How often does s/he keep track of the quantity of sweet drinks (soda/soft drinks, juices) that you drink? (4) |
|  | ¿Con qué frecuencia tus padres revisan la cantidad de bebidas azucaradas que tomas? |
| **Adolescent**  **Control** | This caregiver allows you to eat whatever you wants? (5) |
|  | ¿Tus padres te permiten comer lo que quieras? |
|  | Can you choose the items you want of what is served at luch or dinner, leaving aside what you donor like, without interference from the caregiver? (6) |
|  | ¿Puedes elegir los alimentos que quieres comer de lo que se te sirve al almuerzo o cena, dejando a un lado lo que no te gusta? |
|  | When you do not like what is served for eating, does your caregiver cook something else for you? (10) |
|  | ¿Cuando no te gusta lo que se sirve para comer, ¿tus padres preparan algo más? |
|  | Does this caregiver allow you to have snacks whenever you want? (11) |
|  | ¿Tus padres permiten que comas snacks cuando tú quieres? |
|  | Does this person allow you to leave the table when you are satisfied, even if others have not finished eating? (12) |
|  | ¿Tus padres permiten que dejes la mesa cuando estás satisfecho, incluso si los otros no han terminado de comer? |
| **Restriction for weight control** | This caregiver needs to be sure that I do not eat fatty foods. (18) |
|  | ¿Mis padres necesitan estar seguros de que no como alimentos altos en grasa? |
|  | This person encourages me to eat less food so that I won’t get fat. (24) |
|  | Mis padres me animan a que coma menos comida para que no engorde. |
|  | This person helps me controlling the quantity of food that I serve myself at each meal in order to control my weight. (26) |
|  | Mis padres me ayudan a controlar la cantidad de alimentos que me sirvo en cada comida para controlar mi peso. |
|  | If I eat more than normally at one meal, this person reduces the quantity of food at the next meal. (29) |
|  | Si como más de lo normal en una comida, mis padres reducen la cantidad de alimentos de la siguiente comida. |
|  | This caregiver limits the foods that might make me fat. (30) |
|  | Mis padres restringen la comida que podría hacerme engordar. |
|  | S/he believes that I should not eat certain foods so that I do not gain weight. (31) |
|  | Mis padres creen que no debo comer cierta comida porque me hará engordar. |
|  | I am monitored so that I do not eat between meals in order to not get fat. (36) |
|  | Mis padres no me permiten comer entre comidas para no engordar. |
|  | This caregiver -forces me to restrict my diet in order to control my weight. (39) |
|  | Mis padres me ponen a dieta para controlar mi peso. |
| **Parental**  **Modeling** | This caregiver eats healthy food to give me an example of healthy eating habits. (38) |
|  | Mis padres comen comida saludable para darme un ejemplo de alimentación saludable. |
|  | Even when it is not the caregiver's preferred food, s/he often eats it because s/he finds it important to give me her/his example. (40) |
|  | Incluso cuando no es la comida preferida de mis padres, igual se la comen, porque consideran que es importante. |
|  | S/he tries to show enthusiasm regarding healthy food. (41) |
|  | Mis padres tratan de mostrar entusiasmo respecto a la comida saludable. |
|  | S/he shows me how much s/he enjoys eating healthy food. (42) |
|  | Mis padres me muestran cuanto les gusta comer alimentos saludables. |
| **Environment** | Most of the food at home is healthy. (14) |
|  | La mayoría de la comida en casa es saludable. |
|  | At home, there is a lot of snacks (potato chips, salty popcorn, etc.). **R** (16) |
|  | En casa hay muchos snacks (papas fritas, ramitas, doritos, palomitas de maíz saladas). |
|  | During meal times at home, there are several healthy food items available for me to eat. (21) |
|  | Durante las comidas en casa, hay varios alimentos saludables disponibles para comer. |
|  | At home, there is a lot of sweets (ice cream, cakes, pies, candies/preserves, desserts, goodies). **R** (32) |
|  | En casa, hay muchos dulces (helados, postres, golosinas, queque, caramelos). |

R = Reversed coded
